# Surgical Treatment of Proximal Humerus Unicameral Bone Cyst: A Case Report

**DOI:** 10.7759/cureus.68435

**Published:** 2024-09-02

**Authors:** Mohamed Nasheed, Muhammad Kamal Muhammad Abdul Jamil, Ahmad Fazly Abd Rasid

**Affiliations:** 1 Department of Orthopaedics and Traumatology, Universiti Kebangsaan Malaysia Medical Centre, Kuala Lumpur, MYS

**Keywords:** curettage, humerus, surgical treatment, fracture, unicameral bone cyst

## Abstract

Unicameral bone cysts (UBCs) are noncancerous, fluid-containing sacs commonly seen in the metaphysis of long bones among young individuals, mainly affecting the proximal humerus and femur. Since they are painless, 80% of patients do not experience any symptoms from UBCs unless it is complicated by a pathological or stress fracture. These patients usually present with no history of trauma, with mild pain, local tenderness, and occasionally swelling. The diagnosis of UBCS can either be an incidental finding or can be made with the help of clinical features, radiographs, and differential diagnoses of UBCs like aneurysmal bone cyst, fibrous dysplasia, enchondroma, eosinophilic granuloma, and intraosseous ganglia can be ruled out. While identifying these cysts is often straightforward, there is ongoing debate regarding the optimal management approach. We report a case of a 16-year-old female with proximal humerus UBC who presented with a pathological fracture of the right proximal humerus. The patient was initially managed conservatively. However, she sustained a refracture at the same site twice over four years. Due to fracture recurrence and residual deformity, it was treated surgically with curettage, bone grafting, and internal fixation. The normal alignment and function of the right upper limb were restored postoperatively.

## Introduction

Unicameral bone cysts (UBC) were first recognized by Virchow in 1876 and account for nearly 3% of all bone tumors. Although UBCs, or simple bone cysts, tend to expand and weaken the involved bone, they are not neoplasms in the true sense [1].

Proximal humerus UBCs are benign, lytic lesions primarily observed in the pediatric population, typically affecting individuals between 3 and 14 years of age. These cysts can be incidental findings on imaging studies or can present clinically with pain, swelling, and occasional pathological fractures [2].

Management strategies for UBCs encompass a spectrum of options. Non-surgical approaches, such as observation, steroid injections, and percutaneous procedures, are suitable for small, asymptomatic cysts, particularly those in the upper extremity or calcaneus. Surgical interventions, including curettage and bone grafting, are recommended for symptomatic or recurrent UBCs to decompress the cyst and promote bone healing [3].

The decision for surgical intervention is typically guided by persistent pain, impending or recurrent fractures, and the prevention of secondary deformities. This combined viewpoint highlights the complexity of treating proximal humerus UBCs and emphasizes the significance of individualized treatment plans based on lesion characteristics and patient-specific variables. The main aim of the surgery is to prevent or manage pathological fracture, prevent re-fracture and cyst recurrence, and promote cyst healing [2].

## Case presentation

A 16-year-old female was admitted to the orthopedic department for a right proximal humerus UBC. She was diagnosed with proximal humerus UBC at the age of 10 when she presented with a pathological fracture of the right humerus. She was treated conservatively; however, she sustained a refracture at the same site twice over four years. Due to the recurrence of fracture as well as the residual deformity post-multiple fracture healing at the UBC site, we opted for surgical management.

Physical examination revealed that she had a varus deformity of the right arm. However, no rotational deformity was noted, and the range of motion of the right shoulder and elbow was full. Even though the patient did not complain of pain at the time, there was tenderness on deep palpation at the UBC site. The neurovascular assessment of the right upper limb was unremarkable.

**Figure 1:**
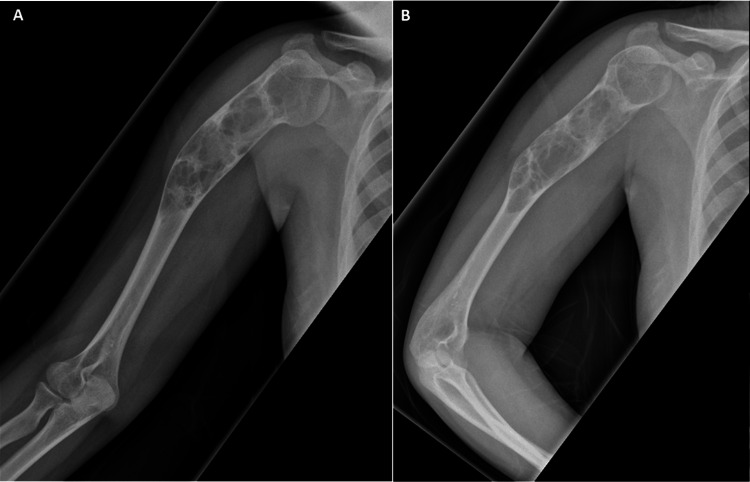
Radiograph of right humerus in anteroposterior (A) and lateral view (B) A: Anteroposterior view, B: Lateral view

A plain radiograph of the right humerus (Figure 1) shows centrally located, well-defined radiolucent lesions with a narrow transition zone and a thin sclerotic margin in the proximal humerus extending to the mid-shaft. There is no periosteal reaction or soft tissue shadow seen; however, there is an expansion of the bone with thinning of the cortex without any breach of the cortex or fracture. It also appears to be in varus deformity in the mid-humerus region.

The patient was positioned supine on the surgical table under general anesthesia. An anterolateral approach to the humerus was performed, extending from the site of the lesion to the middle third of the humerus. The soft tissue was dissected until the humerus bone, and the site of the UBC was visualized. The overlying soft tissue did not have any abnormalities. A bone window was created, and curettage of the UBC was performed. Subsequently, a transdeltoid approach was utilized to expose the proximal aspect of the humerus. A Proximal Humerus Locking System (PHLOS) plate was used, with proximal screws inserted before performing an osteotomy at the site of the cyst to realign the humerus. Distal screws were then inserted, and the cyst was meticulously irrigated before the introduction of a bone graft (30 cc synthetic bone granule). The final placements of the plate, screws, and bone graft were verified with the use of image intensifier guidance. The surgical site was then closed in layers.

**Figure 2:**
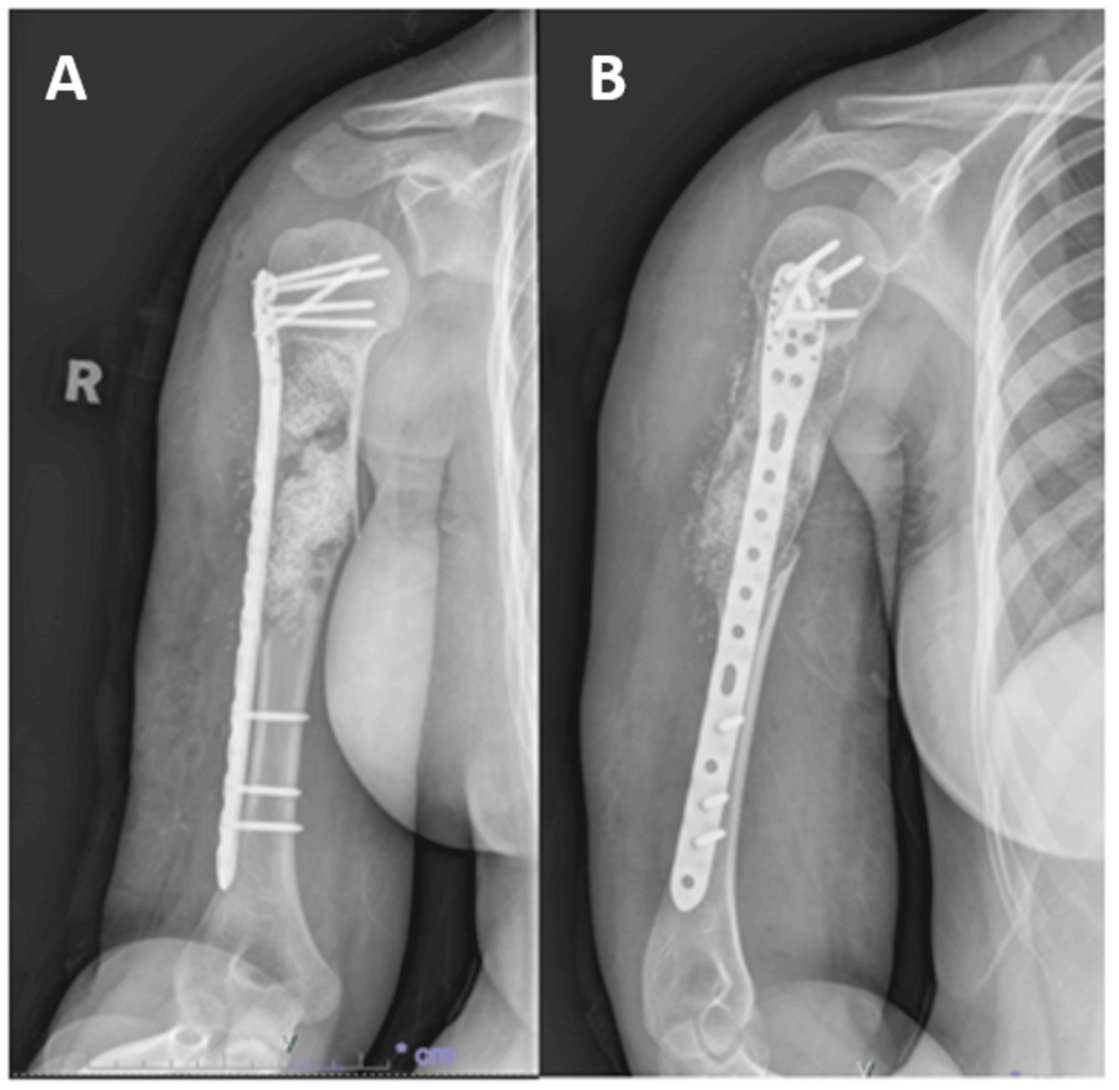
Radiograph of right humerus in anteroposterior and lateral view on postoperative day one A: Anteroposterior view, B: Lateral view

**Figure 3:**
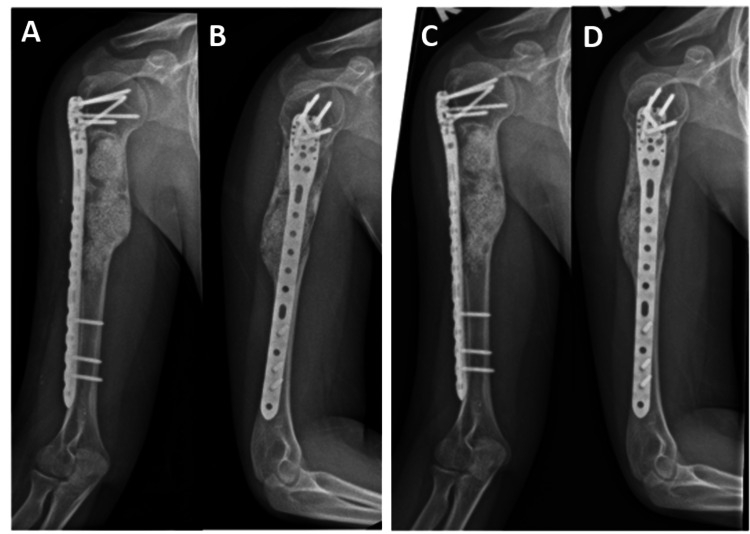
Radiograph of right humerus A: Anteroposterior view three months postoperatively, B: Lateral view three months postoperatively, C: Anteroposterior view six months postoperatively, D: Lateral view six months postoperatively

Postoperative radiographs (Figures 2, 3) demonstrated that the plate and screws were appropriately situated and that the alignment of the humerus was restored.

Postoperatively, the patient’s right upper limb was immobilized in an arm sling for comfort, and pendulum exercise was commenced as early as postoperative day one. Gradually, the range of motion exercises of the right shoulder was changed from passive to active within six weeks postoperatively. Although the patient had some limitations to the range of motion of her right shoulder during the six-week follow-up, it was noted that she had regained full range of motion and power during the three-month postoperative follow-up.

## Discussion

Unicameral bone cysts (UBCs) in the proximal humerus pose challenges in management, with surgical intervention often crucial for achieving resolution and restoring bone integrity. The decision for surgical intervention hinges on several clinical indications supported by current literature. Typically, surgical treatment is considered when UBCs present with symptoms such as pain, swelling, or pathological fractures that compromise bone integrity and functional ability. In this case, the indication for surgery is the recurrent pathological fracture with the residual deformity of the proximal humerus due to multiple fracture healing at the UBC site [4].

The literature emphasizes the significance of surgical management in cases where UBCs cause significant morbidity or functional impairment. Studies indicate that surgical approaches, including curettage with or without bone grafting, are effective in promoting cyst healing and reducing the risk of recurrence compared to non-surgical alternatives [5,6].

Moreover, surgical indications may also consider the location and size of the cyst, with larger cysts or those located in critical anatomical regions warranting more aggressive management strategies to prevent complications and optimize outcomes. While non-surgical options such as observation or minimally invasive techniques may be suitable for asymptomatic or smaller cysts, symptomatic presentations often necessitate surgical intervention to achieve resolution and restore normal bone function [7,8].

The comparative effectiveness of surgical treatments for proximal humerus UBC is a subject of ongoing research aimed at optimizing outcomes and reducing recurrence rates. Various surgical techniques have been described, including bone marrow injection, steroid injection, and percutaneous curettage. The comparison showed that there was a 21%, 41%, and 70% satisfactory healing rate, respectively [3]. This case report highlights a surgical approach involving curettage and bone grafting. Curettage involves meticulous removal of cystic tissue and scraping of the cyst walls to facilitate the infiltration of bone cells and enhance healing. The adjunct use of bone grafts provides structural support and aids in filling the void left by cyst removal. This method is supported by studies demonstrating its efficacy in reducing recurrence rates and improving functional outcomes in UBC patients [4,5].

Various graft materials, including autografts, allografts, and synthetic substitutes, offer options tailored to patient-specific needs and surgical goals [6]. There seem to be no grounds for recommending one filling material over another. The application of electrical or chemical cauterization on the walls of the cyst does not appear to be beneficial. The autologous iliac graft does not seem to provide better results than other methods. Associating drainage with a perforated screw or pin is a useful supplement [7]. Comparative studies emphasize the benefits of surgical intervention over non-surgical approaches, particularly in cases presenting with symptomatic cysts or pathological fractures [8].

While surgical curettage with bone grafting is effective, it is not without risks. Complications such as infection, graft rejection, or injury to surrounding structures emphasize the importance of meticulous surgical technique and postoperative care. Advances in surgical techniques and biomaterials continue to improve treatment strategies, aiming to optimize outcomes and minimize risks associated with UBC management [9,10].

## Conclusions

The significance of surgical interventions, particularly curettage with bone grafting, in managing proximal humerus UBCs is both effective and promising. It is important to guide treatment strategies according to clinical presentations and to support evidence-based practices. Advances in surgical techniques and biomaterials continue to shape UBC management, aiming to optimize outcomes, reduce recurrence rates, and minimize complications associated with these challenging bone lesions.
